# Health professionals’ attitudes to patients’ use of wearable technology

**DOI:** 10.1177/2055207619845544

**Published:** 2019-04-24

**Authors:** Angus Watt, Katherine Swainston, Gemma Wilson

**Affiliations:** 1 Department of Psychology, School of Social Sciences, Humanities and Law, Teesside University, UK; 2 Department of Nursing, Midwifery and Health, Faculty of Health and Life Sciences, Northumbria University, UK

**Keywords:** Wearable technology, biometric monitoring, healthcare, qualitative

## Abstract

**Objective:**

Wearable technologies for health monitoring are becoming increasingly mainstream. However, there is currently limited evidence exploring use from the perspective of healthcare professionals. This study aimed to explore health professionals’ attitudes toward their patients’ use of wearable technologies.

**Methods:**

A convenience sample of health professionals was recruited to participate in this study. Qualitative semi-structured interviews were carried out either face-to-face, via Skype or telephone. Interviews were recorded using a Dictaphone, transcribed verbatim and analysed using thematic analysis.

**Results:**

Four themes emerged from the qualitative findings: ‘opportunities for wearable technology’, ‘usability and understanding’, ‘privacy and surveillance’ and ‘cost’.

**Conclusions:**

The findings portray health professionals’ ambivalence to the use of wearable technology, and it was apparent that whilst the participants considered the technology as being beneficial to patients, they still had concerns for its use.

## Introduction

Wearables can be attached to an individual using a wristband made of functional textiles, or embedded microsystems.^1^ One mainstream wearable is the activity tracker, such as Fitbit, Jawbone and Misfit, which transforms bouts of movement and physical activity into quantitative, measurable data, changing the way in which users understand their own patterns of daily living.^2^ Feedback gained from technology has the ability to motivate individuals to change their behaviour.^3^

It is being increasingly recognised that wearable technology has the potential to transform healthcare, providing care that is distinct from usual medical practice and health delivery.^4^ Innovative wearable technology has the potential to reduce healthcare costs and the pressures faced by frontline healthcare staff,^5^ partly as the result of increased self-care and prevention, increasing individuals’ control of their own health^5^. Particularly, changes to the patient–doctor relationship is cited as being a primarily positive change based on the use of wearables.^6^

Three critical elements of adoption and use have been considered in creating adoption and long-term engagement with wearable technology: habit formation, social motivation and goal reinforcement.^2,7^ However, despite some theoretical consideration of what factors are necessary for the long-term adoption of these technologies, there is currently no evidence examining health professionals’ views of individuals using these technologies. Most of the studies currently exploring the use of wearables in healthcare focus on patient perceptions, some on carers, but few on health professionals’ views.^8–10^ One study explored all three, based on the views of general wearables and a clinically developed wearable used for seizure detection.^9^ Whilst automatic data showing seizure detection during consultation was preferred to non-digital methods, they were worried about increased workload and confidentiality of data, as well as wearability of users.^9^

Whilst much focus of this literature currently rests with the user’s experiences, there is currently little exploration of health professionals’ views recommending digital technologies to patients. This study aimed to explore health professionals’ attitudes toward their patients’ use of wearable technologies.

## Methods

### Design

A qualitative method was employed in which semi-structured interviews were utilised to examine the research aim.

### Participants

A convenience sample of health professionals were recruited to participate in this study. Fliers were sent to general practitioner (GP) surgeries and other health professionals, and personal contacts were informed of the study. Individuals were ineligible to participate only if they were not a practising health professional. A total of 12 health professionals participated in this study, and took part in semi-structured interviews (see Table 1).

**Table 1.** Participant characteristics.

Participant number	Role	Sex	Age	Location	Previous experience of wearable technology
P001	GP	F	49	Edinburgh	None
P002	GP	M	41	Edinburgh	None
P003	Junior doctor	M	24	Glasgow	None
P004	GP	M	45	Edinburgh	None
P005	Retired GP	F	Unknown	Edinburgh	None
P006	GP	M	64	Middlesbrough	Activity tracker
P007	Junior doctor	F	25	Glasgow	None
P008	Personal trainer	F	46	Edinburgh	Activity tracker
P009	Senior nurse	F	53	Edinburgh	None
P010	Junior doctor	M	23	Glasgow	Activity tracker
P011	Physiotherapist	M	24	Dundee	Activity tracker
P012	Occupational therapist	F	27	Glasgow	None

The sample consisted of 12 health professionals; four GPs, three junior doctors, one dietician, one personal trainer/pharmaceutical technician, one consultant nurse, one occupational therapist and one physiotherapist (six males, six females; aged 23–61 years). Only two of the health professionals had previous personal experience using their own wearable technology, but neither used it every day: one participant used an activity tracker whilst running, and one participant used wearable technology whilst skiing.

### Ethical approval

This study received ethical approval from Teesside University's ethical review committee.

### Procedure

**Figure 1.**
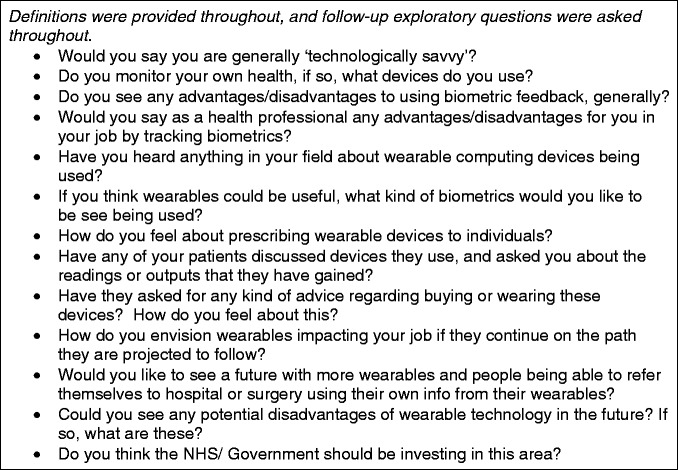
Interview schedule.

The lead researcher (AW) provided individuals with both verbal and written information about the study, including its purpose and details of their participation. Once individuals had been given this information, had the opportunity to answer any questions, and had provided written consent, an appropriate time was arranged to participate in a semi-structured interview. One-to-one semi-structured interviews were either carried out face-to-face, over the telephone, or by using teleconference facilities ensuring that data collection was convenient for the participants involved and widened the locality of participants throughout the United Kingdom (UK). Before the interview began, an example was provided to confirm participants’ understanding of wearable technology (GoBe, HealBe technology™); however, it was stressed that these were only some examples of wearable technology, and all forms of wearable technology were of importance. AW carried out all semi-structured interviews (each lasting between 30 and 60 min) utilising an interview schedule that was informed by relevant literature (see Figure 1). All semi-structured interviews were recorded using a Dictaphone recording device and AW transcribed verbatim. Audacity® software was used to abstract data.

### Data analysis

Data were analysed using inductive Thematic Analysis. Inductive Thematic Analysis aims to extract patterns of data into themes and sub-themes, as they arise from the data.^11^ The six steps of conducting Thematic Analysis were followed, in that the data analyst (AW) familiarised themselves with the data by transcribing and re-reading the data before generating initial codes, searching for themes, reviewing themes, defining and naming themes, before finally producing the report. In order to increase methodological rigour, data analysis was discussed with KS, and themes were checked to ensure they remained representative of the data.

## Results

Four themes emerged from the qualitative findings representing health professionals’ attitudes towards patient us of wearable technology, and their perceptions of implications that this can have on the healthcare service and doctor-patient relationship: ‘opportunities for wearable technology’, ‘usability and understanding’, ‘privacy and surveillance’ and ‘cost’. Whilst the first theme describes the positive aspects that wearable technology can have for individuals, the findings illustrate primarily negative opinions, and the final three themes demonstrate this.

### Opportunities for wearable technology and patient self-management

It was clear that some participants generally felt that patients do not take enough responsibility of their own health and ‘*rely on everyone to sort out the problem’* (P007).

*‘Having a doctor tell you that everything’s looking ok, that’s an easy way out, it’s not up to us, it’s up to you, I think that’s a very negative thing.’* (P002).

There is a sense that health professionals within the sample believe that the public are responsible for looking after themselves and think that they are required only for serious health issues. However, all participants agreed that wearable technologies are a useful tool to promote self-management, enable others to ‘*take responsibility of their own health’* (P007) and ‘*be more aware of themselves’* (P006).

### Usability and understanding

Participants described the importance of usability and of understanding the data recorded by wearable devices. The health professionals had some concerns that some users may not wear the devices due to aesthetics and usability. Namely, there were assumptions that female users may not wear the devices when ‘*dressed up’* (P008) or *‘on a night out’* (P009) if the device was not hidden. Furthermore, participants described usability issues as a potential barrier for use with older adults.

*‘I think that, yeah it's too technical [for older adults]’* (P008).

*‘Even those who are used to smartphone use could see it being too complicated’* (P008).

However, there was recognition that future generations of older adults will be more *au fait* with this technology.

*‘when they go back they didn’t have anything like iPhones or watches or that, whereas, when we are in our eighties we will, so we will be familiar with those things, when our generation get dementia I think stuff like that will be really helpful’* (P012).

Evidently, there were judgements as to who may not use these devices due to usability or aesthetics. Usability issues were also of concern, not for the device itself, but for understanding data provided by the technology, and participants felt that patients may unnecessarily worry about their health either due to data being ‘*misread’* (P010) or by patients over monitoring the data.

*‘I don’t know how effective they would be because you can over monitor, people can become, they think they are unwell when they aren’t’* (P011).

One participant was anxious that constant monitoring of one’s health may ‘*make people more unwell’* (P001). Instead, it was suggested that wearable devices in which data ‘*went straight to a health professional’* (P011) would be more useful. Furthermore, wearable devices are only able to provide some data, which in isolation, may not be enough ‘*to give the full picture’* (P006) about their overall health.

Participants showed concern that patients may worry more about their health due to constant monitoring, trying to make sense of the data presented to them, or misinterpreting the findings. In essence, there seems to be a need for user education, knowledge and understanding of the devices.

### Privacy and surveillance

Privacy is a much-debated aspect of technology use. The importance of suitable and reliable measures in place for individual’s health information is of upmost importance, especially when being used with relation to healthcare services.

*‘I don’t know, I suppose it’s kinda ethically, is acceptable to constantly know what someone’s health biometrics are’* (P011).

Participants referred to wearable technology as *‘a silent data gathering thing’* (P003) and as ‘*big brother’* (P012).

*Ethical as well as privacy concerns are going to need to be certified and new laws are going to be required. ‘Privacy, sharing of the information, that sort of thing, would be, all that would have to be clarified’* (P004).

Concerns of privacy are of obvious worry to participants and are further compounded by both research and policy lagging behind continuously emerging and changing invocations.

### Cost

The ability to save money is a huge concern for the National Health Service (NHS) and for those that work within it. Participants discussed both the potential long-term cost savings to healthcare systems due to increased autonomy, self-management of long-term conditions, and improved preventative behaviours.

*‘if you can prevent a fall happening, each operation costs £10,000 pounds so if you can prevent an operation happening, you're saving the NHS a lot of money’* (P011).

*‘You’d save the NHS a lot of money. I think that is a big question, yeah, I think there are things you can do, and most of them are to do with motivation’* (P010).

However, whilst some considered long-term cost saving to the NHS to be a result of reduced emergency admission to hospital and improved methods of self-management, some also discussed the barriers to providing patients with these devices over traditional treatments, such as drugs.

*‘I suppose the issue for the NHS is the cost for these things’* (P011).

There were two ways of considering cost of wearable devices: as potentially saving NHS funds in the long-term, but also the short-term cost of the actual devices.

## Discussion

This study aimed to explore health professionals’ attitudes toward patients’ use of wearable technologies. Four themes were generated from the interview data collected: opportunities for wearable technology, usability and understanding, privacy and surveillance and cost. The themes illustrate the ambivalent views of health professionals involved in this study, and it was apparent that whilst the participants considered the technology as being beneficial to patients, they still had concerns for its use. The health professionals understood there was a use for wearables but could see a number of limitations that would require to be rectified before mass adoption.

An unexpected discovery from the current study was the realization that those health professionals interviewed did not think the NHS was adequately providing preventive care to avoid illness or disease. Most interviewees agree that the NHS was instrumental at administering life-threatening interventions and ongoing chronic cases; however, they stressed that consultation time was taken up as a result of the lack of focus of preventative care and the self-management of long-term conditions in the NHS. Similar to the developing health policy in the UK,^5^ health professionals felt that wearable technologies could be used as tools to improve illness and disease prevention, and the self-management of long-term conditions.

Barriers to use included gendered and age-related perceptions of aesthetic design, usability and interpretation, which were discussed. All of which have been considered in previous research primarily by the user’s own perspective but also by the perspective of health professionals.^9^ The aesthetics of technology are important, and are considered in general models of technology use and user experience and empirical research which evidence the importance of wearability,^12^ including aesthetics and comfort.^13–16^ Interestingly, within this study, health professionals’ perceptions of the importance of aesthetics impact their recommendation of wearable technology to patients. Furthermore, it is apparent that the health professionals in this study consider age to be a barrier. Although it is important to consider age-related aspects of technology design and usability such as sensory, motor and cognitive functioning,^17,18^ evidence has demonstrated many pieces of wearable technology as being suitable for use, and enjoyed, by older adults.^19,20^ Some studies focusing on older adults’ experiences of wearable technology, show that preconceptions make individuals more wary of the technology,^19^ often with older adults considering it to be designed for younger people.^21^ However, interestingly, older adults within one study specifically stated that promotion of these wearable devices by health professionals would make them more likely to use them.^21^

Wearables relating to healthcare must be considered with high privacy concerns,^22^ and this was clearly an important consideration for participants in this study, reflecting previous research.^9^ Data protection concern has been growing but an area of heated debate is in the medical sphere. This is also an area of considerable concern for the NHS, and is a central point of its digital innovation plans, to ensure ‘*every citizen’s data is protected*’.^23^ Furthermore, cost was a concern, and participants not only considered the cost savings but also the cost and sustainability of these devices within NHS budget.

This study presents some limitations. Primarily, the sample size was small and consisted of health professionals primarily from one region. Differing usage and experiences may be observed with different groups of health professionals and cultural factors may also play a role. Furthermore, the researcher had previous personal connections with some participants, which may have impacted their participation and response. The study remit was broad, including any wearable technology, and, accordingly, exploring the use of specific wearable technologies may be informative. Future research must build on these findings and provide a more comprehensive evidence base in this area, as policy is promoting the uptake of wearable use for prevention and self-management; however, the importance of adopting wearable technologies also lies with health professionals.

## References

[1] Tröster G. The agenda of wearable healthcare. Yearb Med Inform 2005; 14: 125–138. 27706307

[2] Gilmore JN. Everywear: The quantified self and wearable fitness technologies. New Media Soc 2015; 11: 2524–2539.

[3] Western MJ Peacock OJ Stathi A et al The understanding and interpretation of innovative technology-enabled multidimensional physical activity feedback in patients at risk of future chronic disease. PloS one 2015; 10: e0126156. 25938455 10.1371/journal.pone.0126156 PMC4418766

[4] Korhonen I Pärkkä J Van Gils M. Health monitoring in the home of the future. IEEE Eng Med Biol Mag 2003; 22: 66–73. 10.1109/memb.2003.1213628 12845821

[5] National Information Board. Personalised Health and Care 2020: Using data and technology to transform outcomes for patients and citizens, a framework for action. London: NHS, 2014.

[6] Lupton D. The digitally engaged patient: Self-monitoring and self-care in the digital health era. Soc Theory Health 2013; 11: 256–270.

[7] Ledger D McCaffrey D. Inside wearables: How the science of human behavior change offers the secret to long-term engagement. *Endeavour Partners, https://medium.com/@endeavourprtnrs/inside-wearable-how-the-science-of-human-behavior-change-offers-the-secret-to-long-term-engagement-a15b3c7d4cf3* (2014, accessed 11 June 2018).

[8] Asimakopoulos S Asimakopoulos G Spillers F. Motivation and user engagement in fitness tracking: Heuristics for mobile healthcare wearables. Informatics 2017; 4: 5.

[9] Bruno E Simblett S Lang A et al Wearable technology in epilepsy: The views of patients, caregivers, and healthcare professionals. Epilepsy Behav 2018; 85: 141–149. 29940377 10.1016/j.yebeh.2018.05.044

[10] Piwek L Ellis DA Andrews S et al The rise of consumer health wearables: Promises and barriers. PLoS Medicine 2016; 13: e1001953. 26836780 10.1371/journal.pmed.1001953 PMC4737495

[11] Braun V Clarke V. Using thematic analysis in psychology. Qual Res Psychol 2006; 3: 77–101.

[12] Hassenzahl M Tractinsky N. User experience – a research agenda. Behav Inform Technol 2006; 25: 91–97.

[13] Gemperle F Kasabach C Stivoric J et al Design for wearability. In: Digest of Papers. Second International Symposium *on* Wearable Computers, Pittsburgh, PA, 19–20 October *1998*, pp.116–122. Piscataway, NJ: IEEE.

[14] Knight JF Baber C Schwirtz A et al The Comfort Assessment of Wearable Computers. In: *Proceedings. Sixth International Symposium on Wearable Computers*, Seattle, WA, 10 October 2002, pp. 65–74. Piscataway, NJ: IEEE.

[15] Park S Jayaraman S. Enhancing the quality of life through wearable technology. IEEE Eng Med Biol Mag 2003; 22: 41–48. 12845818 10.1109/memb.2003.1213625

[16] Miner CS Chan DM Campbell C. Digital jewelry: Wearable technology for everyday life. In: CHI'01 extended abstracts on Human factors in computing systems, pp. 45–46. New York: ACM, 2001.

[17] Chen K Chan A. A review of technology acceptance by older adults. Gerontechnology 2011; 10: 1–12.

[18] Rogers WA Mynatt ED. How can technology contribute to the quality of life of older adults. In: M.E. Mitchell (eds) *The technology of humanity: Can technology contribute to the quality of life* Chicago: Illinois Institute of Technology, 2003: pp. 22–30.

[19] Wilson G Jones D Schofield P et al Experiences of using a wearable camera to record activity, participation and health-related behaviours: Qualitative reflections of using the Sensecam. DIGITAL HEALTH 2016. DOI: 10.1177/0141076817700848. 10.1177/2055207616682628 PMC6001196 29942578

[20] Lyons EJ Swartz MC Lewis ZH et al Feasibility and acceptability of a wearable technology physical activity intervention with telephone counseling for mid-aged and older adults: a randomized controlled pilot trial. JMIR Mhealth Uhealth 2017; 5: e28. 28264796 10.2196/mhealth.6967 PMC5359416

[21] Mercer K Li M Grindrod KA. Do wearable activity trackers have a place in pharmacies? Can Pharm J (Ott) 2015; 148: 134–137. 26150885 10.1177/1715163515579221 PMC4483759

[22] Gao Y Li H Luo Y. An empirical study of wearable technology acceptance in healthcare. Ind Manage Data Syst 2015; 115: 1704–1723.

[23] NHS Digital, https://digital.nhs.uk/ (2017, accessed 10 May 2018).

